# Diagnosis of Endophthalmitis and Orbital Abscess by Ultrasound: A Case Report

**DOI:** 10.5811/cpcem.1427

**Published:** 2023-11-08

**Authors:** Stephen Haight, Srikar Adhikari

**Affiliations:** * University of California San Francisco, Fresno Medical Education Program, Department of Emergency Medicine, Fresno, California; † University of Arizona, Department of Emergency Medicine, Tucson, Arizona

**Keywords:** *case report*, *ultrasound*, *endophthalmitis*, *orbital abscess*

## Abstract

**Introduction:**

The diagnosis of ocular pathology by point-of-care ultrasound (POCUS) has been well established for entities such as retinal detachment, vitreous hemorrhage, posterior vitreous detachment, and lens dislocation.^1^ However, the use of ultrasound to detect other conditions such as orbital abscess and endophthalmitis in the emergency setting is rarely reported.

**Case Report:**

We present a case in which POCUS was used to confirm the suspected diagnosis of endophthalmitis and orbital abscess. This case report will review the ultrasonographic findings of orbital abscess and endophthalmitis, as well as briefly discuss the literature for the use of ultrasound for these applications.

**Conclusion:**

Point-of-care ultrasound can be used to rapidly diagnose infectious pathology of the eye and orbit, which could potentially decrease time to diagnosis and time to consultation of these vision-threatening pathologies.

Population Health Research Capsule What do we already know about this clinical entity?

*Orbital abscess and endophthalmitis are uncommon vision-threatening infections that require timely intervention for good outcomes.*

What makes this presentation of disease reportable?

*The co-occurrence of orbital abscess and endophthalmitis in the same patient is a rare and complex presentation of these serious eye infections.*

What is the major learning point?

*Point-of-care ultrasound can rapidly diagnosis both orbital abscess and endophthalmitis, which can lead to earlier IV antibiotics and surgical consultation.*

How might this improve emergency medicine practice?

*Recognizing the ultrasound findings of orbital abscess and endophthalmitis would improve time to diagnosis and treatment.*

## INTRODUCTION

Infections of the orbit and periorbital region are common ophthalmic emergencies resulting in significant local and systemic morbidity. Loss of vision occurs in approximately 10% of patients, and systemic complications can include meningitis, intracranial abscess, sepsis, and death.^2^ Rapid diagnosis of orbital infections is crucial to prevent complications and improve patient outcomes. Clinical examination is not always reliable to distinguish pre-septal pathology from other serious vision-threatening conditions. The utility of point-of-care ultrasound (POCUS) to diagnose entities such as retinal detachment, vitreous hemorrhage, posterior vitreous detachment, and lens dislocation has been well established.^1^ However, the use of ultrasound for the diagnosis of other conditions such as orbital abscess and endophthalmitis in the emergency department (ED) setting has been rarely described.^3^ ^–^ ^5^ This case illustrates the ultrasonographic findings of orbital abscess and endophthalmitis, and the role of ultrasound in the evaluation of the patients presenting to the ED with these conditions.

## CASE REPORT

A 52-year-old female with past medical history of hypertension, type 2 diabetes mellitus, hypothyroidism, and bilateral cataract surgery was transferred to our ED for further evaluation. She initially presented to the outside ED for seven days of worsening right eye pain, redness, swelling, and worsening vision, which progressed to complete right-sided vision loss five days prior to her initial presentation. She also noted three days of fever but no recent symptoms of upper respiratory infection, dental infection, eye trauma, or recent surgeries. Vital signs were unremarkable. On examination, the right periorbital soft tissues were significantly swollen and erythematous with chemosis of the conjunctiva, cloudy anterior chamber, and exophthalmos of the right eye. Right eye intraocular pressure was 40 millimeters of mercury (mm Hg) (normal 10–21 mm Hg) with painful extraocular movements. The left eye was normal.

**Image 1.**
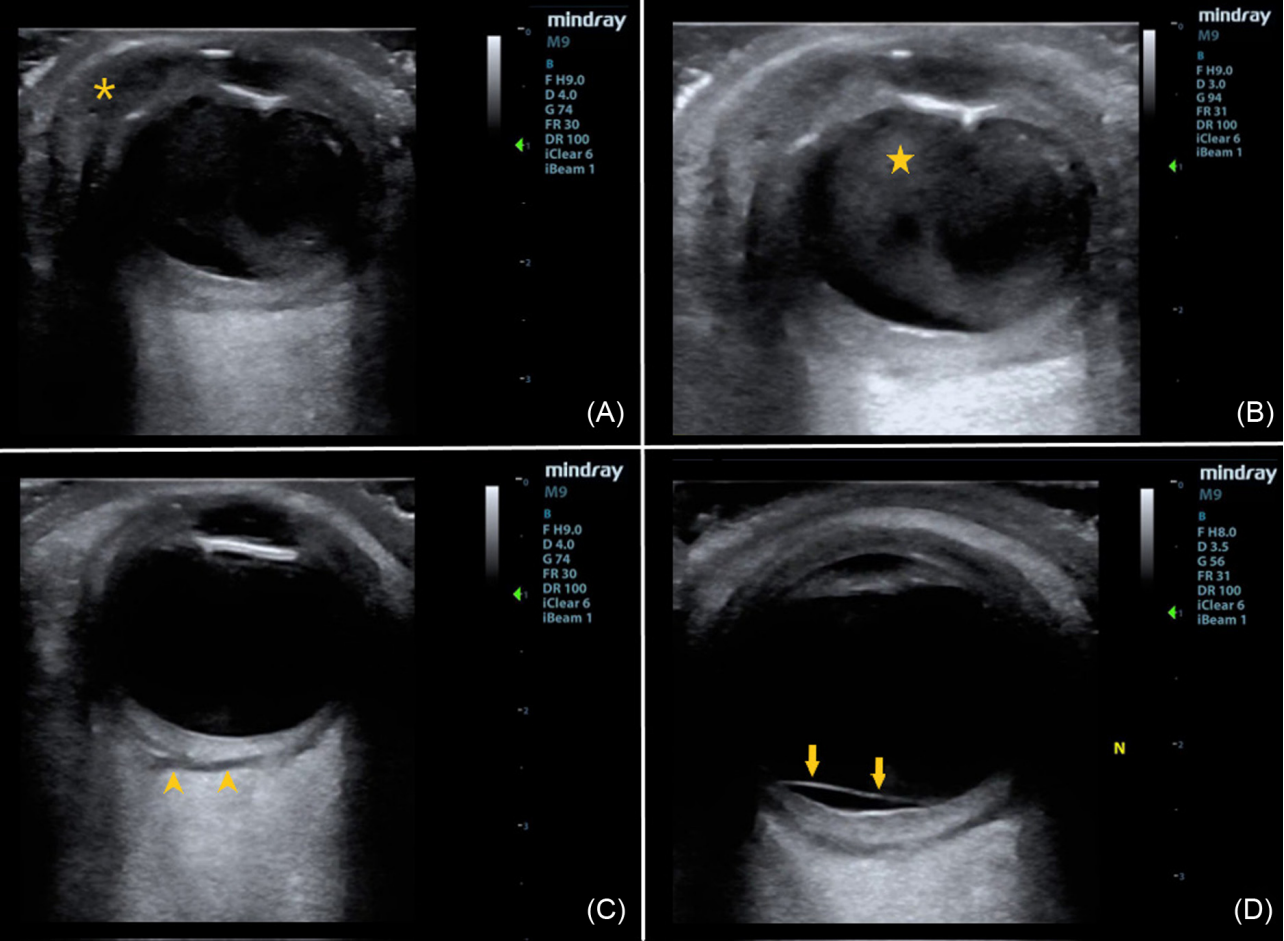
(A) The hypoechoic fluid collection (asterisk) in the lateral aspect of the orbit is consistent with orbital abscess. (B) The echogenic purulent debris within the vitreous humor (star) has an irregular and loculated appearance and is the key ultrasonographic finding of endophthalmitis. (C) and (D) The hypoechoic region posterior to retina and choroid (arrowheads) represents fluid in sub-Tenon’s space (potential space), and the thin membrane crossing the optic nerve (arrows) represents a posterior vitreous detachment, both of which are less common ultrasonographic findings of endophthalmitis.

**Image 2.**
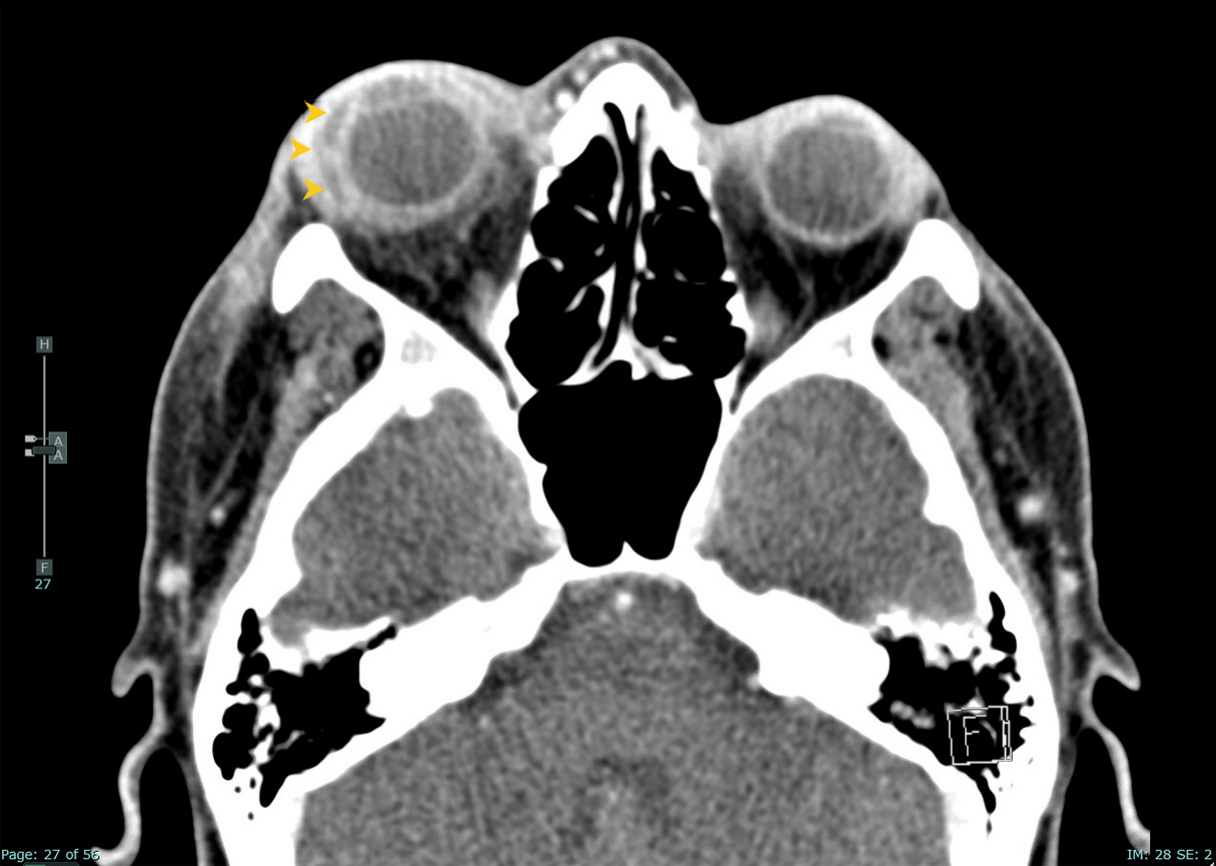
Rim-enhancing fluid collection (arrowheads) lateral to the right globe consistent with orbital abscess.

Point-of-care ultrasound performed in the ED showed loculated echogenic material within the vitreous humor, posterior vitreous detachment, and fluid in sub-Tenon’s space (potential space between the capsule of the eye and choroid) consistent with endophthalmitis. It also showed soft tissue thickening of the eyelid, edema in the orbital fat, and a hypoechoic fluid collection next to the lateral aspect of the globe consistent with orbital cellulitis and orbital abscess (Image 1, Video 1). An orbital computed tomography (CT) demonstrated a right orbital abscess (18 mm × 5 mm × 12 mm) along the lateral and inferolateral margin of the right orbit with preseptal and postseptal cellulitis without osseous involvement (Image 2).

Ophthalmology was consulted and a bedside intraocular aspiration and intravitreal injection of antibiotics was performed. The patient was admitted to internal medicine for broad-spectrum antibiotics. Her intravitreal aspirate grew coagulase negative staphylococcus, and the inciting event was thought to be syphilitic uveitis and vitritis complicated by staphylococcal infection given her positive syphilis serology. The patient did not have any other physical findings of secondary or tertiary syphilis, such as rash or gummas. She was discharged on hospital day 12 on three weeks of intravenous (IV) ceftriaxone and vancomycin and two weeks of oral metronidazole. She was scheduled for a vitrectomy at another facility.

## DISCUSSION

Rapid diagnosis of endophthalmitis and/or orbital abscess is paramount due to the vision-threatening nature and risk for intracranial extension of infection. The most common ultrasonographic findings of endophthalmitis include a moderate to severe quantity of echogenic debris in the vitreous humor and vitreous membranes with loculations.^6^ ^,^ ^7^ Less common ultrasonographic findings include retinal or choroidal detachments, which are present in 7–20% of cases of endophthalmitis but portend worse visual outcomes.^6^ ^,^ ^7^ Other less common findings include choroidal thickening, macular edema and optic disc edema, posterior vitreous detachment, and fluid in sub-Tenon’s space.^6^ ^,^ ^7^ The diagnostic accuracy of ultrasound of these findings is not well established, but small, non-randomized studies suggest ocular ultrasound has a sensitivity of >90% and specificity of >79% for the diagnosis of endophthalmitis.^8^ ^,^ ^9^

Ultrasonographic findings of orbital cellulitis include hyperechoic inflammatory intraconal fat, heterogenous collection of intraorbital material with mixed echogenicity (hyperechoic and hypoechoic), and extraocular muscle edema and/or displacement.^4^ ^,^ ^10^ ^–^ ^12^ Orbital abscess typically appears as an anechoic to hypoechoic fluid collection, but as with soft tissue abscesses elsewhere in the body, they can also appear isoechoic and contain hyperechoic debris.^4^ ^,^ ^10^ ^–^ ^12^

The diagnostic accuracy of ultrasound of these findings of orbital cellulitis and abscess has not been well established, but small non-randomized studies suggest ocular ultrasound has a potential for high diagnostic accuracy.^13^ ^,^ ^14^ Computed tomography is more sensitive for diagnosing orbital abscess than ultrasound and will assist with operative planning. However, there are several situations in which ultrasound can be particularly helpful in the evaluation of infections of the eye and orbit. Firstly, POCUS can be performed rapidly at bedside, leading to earlier surgical consultation and hospital admission for administration of IV antibiotics, both of which could potentially lead to better outcomes for these vision-threatening conditions. Additionally, ultrasound can help provide important clinical information beyond what can be obtained in the history and physical examination alone, especially in cases without a clear-cut diagnosis, and the clinician is debating the necessity of additional imaging with CT. For example, a patient may have trouble differentiating eye pain from eyelid edema and inflammation versus pain from extraocular movements.

Objective findings of deeper infection on ultrasound would indicate the need for a CT scan in cases that lack obvious physical exam findings of orbital cellulitis, such as proptosis and ophthalmoplegia. In these indeterminate cases, a point-of-care ultrasound showing only superficial eyelid edema would potentially increase the confidence in the diagnosis of periorbital cellulitis and the appropriateness of outpatient management with oral antibiotics which would lead to decreased cost, decreased ED length of stay, and avoidance of ionizing radiation exposure to the patient from CT. Lastly, in resource-limited settings lacking CT availability, ultrasound could help guide appropriate management toward transfer of the patient to a CT-capable facility.

## CONCLUSION

This patient’s case of simultaneously diagnosed endophthalmitis and orbital abscess is a rare presentation of ocular/orbital infection. It highlights the potential for POCUS to rapidly diagnose non-traumatic infectious pathology of the eye as well as lead to early ophthalmology consultation and appropriate treatment of these vision-threatening disease processes.

## Supplementary Information

Video 1. Short cine clip (1/4x speed) demonstrating fluid in sub-Tenon’s space, echogenic purulent debris within the vitreous humor, and posterior vitreous detachment, all of which are potential findings of endophthalmitis.
